# Quality of life and asthma control in pregnant women with asthma

**DOI:** 10.1186/s12890-021-01797-9

**Published:** 2021-12-17

**Authors:** Nasrin Fazel, Michael Kundi, Erika Jensen-Jarolim, Isabella Maria Pali-Schöll, Asghar Kazemzadeh, Habibollah Esmaily, Mojtaba Fattahi Abdizadeh, Roya Akbarzadeh, Raheleh Ahmadi, Hossain Jabbari

**Affiliations:** 1 Iranian Research Center On Healthy Aging, Department of Medical-Surgical Nursing, School of Nursing and Midwifery, Sabzevar University of Medical Sciences, Sabzevar, Iran; 2 Department of Environmental Health, Center for Public Health, Medical University Vienna, Vienna, Austria; 3 Institute for Pathophysiology and Allergy Research, Medical University Vienna, Vienna, Austria; 4 Comparative Medicine, The Interuniversity Messerli Research Institute of the University of Veterinary Medicine Vienna, Medical University Vienna and University of Vienna, Vienna, Austria; 5 Department of Biostatistics and Epidemiology, Neonatal Research Center, Mashhad University of Medical Sciences, Mashad, Iran; 6 Department of Anesthesia and Operating Room, School of Paramedic, Sabzevar University of Medical Sciences, Sabzevar, Iran; 7 Tehran University of Medical Sciences, Tehran, Iran

**Keywords:** Asthma severity, Asthma control, Quality of life, Pregnancy

## Abstract

**Background:**

Asthma is the most commonly occurring respiratory illness during pregnancy. Associations with complications of pregnancy and adverse perinatal outcome have been established. However, little is known about quality of life (QoL) in pregnant women with asthma and how it relates to asthma control particularly for Iran.

**Objective:**

To determine the relationship between asthma related QoL and asthma control and severity.

**Methods:**

We conducted a prospective study in pregnant women with asthma. We used the Asthma Control Questionnaire and the Asthma Quality of Life Questionnaire (AQLQ) and the guidelines of the Global Initiative for Asthma for assessment of asthma severity.

**Results:**

Among 1603 pregnant women, 34 were diagnosed with asthma. Of these 13 had intermittent, 10 mild, 8 moderate and 3 severe persistent asthma. There was a significant decrease of QoL with poorer asthma control (*p* = 0.014). This decline could be due to limitations of activity in those with poorer asthma control, which is underlined by the significant decline of QoL with increasing asthma severity (*p* = 0.024).

**Conclusion:**

Although the majority of pregnant women with asthma had a favorable score in AQLQ, reduced QoL was related to increased asthma severity and poor asthma control. This underlines the importance of controlling asthma during pregnancy not only for the prevention of adverse pregnancy outcomes but also for the preservation of QoL.

## Introduction

Asthma is a chronic inflammatory disease of the airways. This inflammation leads to recurrent episodes of coughing, chest tightness, wheezing, and /or shortness of breath that can affect the patient’s performance and quality of life [1]. More than 300 million people worldwide suffer from asthma, a figure expected to rise to 400 million by 2025 [2]. If insufficiently treated, asthma may lead to death. Annual mortality is estimated at 250,000 cases worldwide [2].

Between 8 and 13% of pregnant women have asthma [3, 4]. Asthma may affect quality of life especially in pregnancy; it has an impact on family life, social activities and limits the patients' physical activity thereby adversely affecting women’s daily life during pregnancy [1]. The goal of health care for patients with chronic diseases is not only to reduce mortality but also to improve quality of life [5].

Asthma is influenced by several factors which can either aggravate or ameliorate the condition, and hence the patient’s quality of life [6]. Asthma-specific quality of life (QoL) reflects the individual’s experience with asthma beyond the actual symptoms and is separate from objective data such as pulmonary function. Therefore, the asthma QoL may be a more subtle indicator of deficiencies, effects, and threats from asthma during gravidity than actual symptoms and pulmonary function data [7]. Since asthma is one of the most frequent chronic diseases in pregnancy, it is important to study the quality of life of these patients. By determining the quality of life, treatments could be adjusted to address the specific limitations associated with the illness [8, 9]. Fonseca et al. showed that quality of life decreased with increasing severity of asthma [10]. Mancuso et al. observed that symptoms of depression in patients with asthma are related to lower quality of life [11]. However, quality of life during pregnancy is in a sense unique, because the treatment must address both the mother’s illness and effects on the developing fetus.

This study aims to assess whether asthma control and severity are associated with quality of life in pregnant women in Iran.

## Material and methods

A prospective study in pregnant women with and without asthma that visited the antenatal clinic at Mobini Hospital during the end of the first trimester of their pregnancy was conducted from August 2014 to June 2015. Written informed consent was acquired from study participants. The study was approved by the Research Ethics Committee of Iran (Medsab Rec. 93.36).

All women who fulfilled the following inclusion criteria were eligible: Pregnant women referred to Mobini hospital (the only hospital in the city that performs deliveries) who were expected to deliver in this hospital and who also had their prenatal care at the Mobini hospital or another city clinic that were before the 24th week of gestation. Pregnant women were recruited on average during the 12th weeks of gestation.

Assignment to the group of women with asthma was based on diagnosis of asthma. All women with suspected asthma were transferred to a pulmonologist, specialized in asthma, and a spirometry was performed. Follow-up interviews were managed by phone at 20, 28, and 36 weeks (± 5 days) of gestation and in the hospital following childbirth. Patients were classified with respect to clinical severity as per GINA guidelines, using frequency of symptoms and lung function assessed according to the guidelines of the American College of Obstetricians & Gynecologists (ACOG) and the American College of Asthma, Allergy & Immunology (ACAAI). Patients were divided into two groups: intermittent asthma and mild persistent asthma (IMPA), moderate and severe persistent asthma (MSPA) [12].

Women with asthma, depending on the severity of asthma and individual needs, visited the asthma specialist clinic several times during pregnancy. They were divided applying the 5-item version of the Asthma Control Questionnaire (ACQ) [13]. Patients were asked to recall their experiences during the previous week and to respond to the questions on a 7-point scale (0 = no impairment; 6 = maximum impairment). Questions were about nighttime waking, symptoms on waking, activity limitation, shortness of breath, and wheeze. Scores are means across the 5 items. A score of 0.0–0.75 is classified as well-controlled asthma; 0.75–1.5 as a ‘grey zone’; and > 1.5 is classified as inadequately controlled asthma [14]. We used a dichotomy with a cut point of 0.75 (well controlled vs. partly/poorly controlled asthma) from the time point of the administration of the Asthma Quality of Life Questionnaire (AQLQ). All asthma patients were requested to do self-monitoring peak flow measurements and to record these in a logbook for 2 weeks after each asthma specialist clinic visit or after a worsening of symptoms. Inhaled glucocorticoids and any other drug intake were recorded for each trimester during pregnancy.

The AQLQ is a 32-item self-administered questionnaire with four domains (specific functional areas of concern): symptoms (12 questions), activity limitations (11 questions), emotional function (5 questions), and environmental stimuli (4 questions) [15]. Patients responded to each question based on their experiences through the previous two weeks. Responses for each question were on a 7-point Likert scale with scores ranging from 1 (worst; severely impaired) to 7 (best; not impaired at all). Domains scores were obtained as the mean score of the items in that domain. The overall score is computed as the mean of all 32 AQLQ items. The mean was transformed into a scale ranging from 0 to 100, with higher values indicating better quality of life in order to facilitate interpretation as often applied for quality-of-life questionnaires (e.g. WHOQOL). The AQLQ was translated into Persian and then back translated into English by two professional translators. Then, the content validity of the questionnaire was approved by a group of specialists. The final Persian version was filled in by 40 asthmatic patients in Sabzevar, to determine its internal consistency (Cronbach's alpha), which was calculated as 0.86. The AQLQ was administered once in the 2^nd^ trimester.

The study had as main objective the assessment of the relationship between asthma and complications of pregnancy and birth [16], as second objective quality of life in pregnant women with asthma should be assessed in relation to asthma severity and control. Sample size was determined based on a preliminary study that estimated a prevalence of asthma in pregnancy of 5%. However, it turned out that asthma prevalence was well below that expected value, which reduced the power of the study. Concerning the secondary objective, focusing on QoL measured by the AQLQ, power considerations were based on the following assumptions: standard deviation of scores close to 1.0 (~ 17 for the transformed scale), a difference of 1 considered as relevant, and a significance level of 5%. Under these assumptions, for the non-parametric test the study had a power of 75%. Data were analyzed using SPSS 23 (IBM Corp., USA). Women with asthma were compared with those without concerning sociodemographic and other characteristics by Fisher’s exact probability test for binary variables, by chi-square tests for categorical, and by Mann-Whiney U tests for continuous variables. Comparisons within the group of women with asthma according to different characteristics were done by Mann–Whitney U tests except when one group had 5 or less members, in which case Student’s t tests were performed. Multivariable linear regression analysis was performed to assess the relationship between AQLQ domains and asthma severity and asthma control. Age, ethnicity, area of living and parity were considered as confounders. For all tests a 5% significance level was chosen.

## Results

Overall, 1603 pregnant women were enrolled with evaluable data and 34 were diagnosed with asthma (asthma prevalence of 2.1%). Pregnant women with asthma were about 4 years older on average than women without asthma (*p* = 0.001), and therefore were more often multipara (*p* = 0.017) and had more previous spontaneous abortions (*p* = 0.033). They were more frequently of Turk origin (*p* < 0.001), lived more often in a village (*p* = 0.014) and were less educated (*p* = 0.029).

No statistically significant differences were found for weight gain during pregnancy and for weeks of gestation at birth (delivery). More than one quarter of women with asthma had a family history of asthma and almost all of them (94%) had a history of allergies (Table 1). The non-asthmatic group was included here to show the prevalence of asthma and to provide information about the characteristics of women with asthma in contrast to women without.

**Table 1:** Sociodemographic and obstetric characteristics of asthmatic and non-asthmatic women (percentages or means ± standard deviation)

Variable	Non-asthmatic women (n = 1569)	Asthmatic women (n = 34)	*p* value
Age (years)	27.3 ± 5.9	31.0 ± 6.4	0.001^1^
*Education*			
Illiterate	1.6%	5.9%	0.029^2^
Elementary	19.7%	29.4%	
Pre-high school	21.4%	29.4%	
high school/college	57.3%	35.3%	
Employed	5.5%	8.8%	0.435^3^
*Ethnicity*			
Farsi	84.3%	55.9%	< 0.001^2^
Turk	14.2%	44.1%	
Other	1.5%	0%	
*Living in*			
City	68.5%	47.1%	0.014^3^
Village	31.5%	52.9%	
Family history of asthma	1.0%	26.5%	< 0.001^3^
Any allergy	12.7%	94.1%	< 0.001^3^
Previous stillbirth(s)	1.6%	2.9%	0.430^3^
Previous abortion(s)	21.5%	38.2%	0.033^3^
*Parity*			
1^st^	40.7%	26.5%	0.017^2^
2nd	37.4%	29.4%	
3^rd^	17.3%	32.4%	
> 3rd	4.5%	11.8%	
Weight gain (kg)	12.1 ± 4.4	12.6 ± 5.7	0.866^1^
Gestational age at delivery(weeks)	38.9 ± 1.9	39.4 ± 1.2	0.339^1^

^1^Mann–Whitney U test; ^2^ Chi-square test; ^3^ Fisher’s exact probability test

Overall, 11 (32%) women with asthma had MSPA, while 23 (68%) had IMPA. Thirteen women (38%) had well-controlled asthma, and 21 (62%) had partly/poorly controlled asthma.

Favorable quality of life (QoL), defined as a score above 75 [17], was found for the overall score in 2 women only, for the symptoms and activity domains 4 women were in the favorable range, in the emotional domain it were 6 women and for the domain ‘environmental stimuli’ 11 women. Although very good QoL was rare, 87% of women with asthma had scores above 50, considered as at least partly favorable conditions.

### Quality of life and sociodemographic characteristics

Pregnant women with asthma living in villages had significantly lower symptoms related QoL (*p* = 0.025) and a tendency for lower overall QoL (*p* = 0.081). No large differences were observed between ethnicity groups but a tendency for higher symptoms and activity related QoL in Farsi women was noted. No differences in asthma related QoL were found between age and parity groups. Hospital admission of the mother during pregnancy up to the time of QoL assessment did not show an effect on QoL. However, women whose newborn was later admitted to a neonatal ward had much higher emotional QoL (*p* = 0.002) and even overall asthma related QoL was higher (*p* = 0.025) (Table 2).

**Table 2:** Median (and interquartile range) of asthma quality of life questionnaire (AQLQ) domain scores and total score in relation to sociodemographic characteristics, parity, hospital admission of mother and child after pregnancy in women with asthma. *p* value from Mann–Whitney test^1^

Variable	Category	n (%)	Symptoms	Activities	Emotional	Environmental stimuli	Total
Living in	City	16 (47.1)	60.7 (53.6–72.6)	70.1 (66.6–71.8)	58.6 (53.6–67.1)	76.8 (62.5–85.7)	67.6 (60.6–69.9)
	Village	18 (52.9)	53.6 (39.6–64.6)	63.6 (56.5–70.8)	54.3 (37.9–67.1)	75.0 (67.9–84.8)	58.0 (52.3–68.3)
			*p* = 0.025	*p* = 0.154	*p* = 0.297	*p* = 0.878	*p* = 0.081
Ethnicity	Turk	15 (44.1)	53.6 (39.9–63.1)	62.3 (54.5–70.1)	57.1 (45.7–77.1)	75.0 (62.5–85.7)	60.3 (50.2–68.8)
	Farsi	19 (55.9)	60.7 (53.6–70.2)	70.1 (64.3–73.4)	57.1 (51.4–62.9)	75.0 (66.1–83.9)	64.7 (58.0–70.1)
			*p* = 0.077	*p* = 0.083	*p* = 0.811	*p* = 0.864	*p* = 0.202
Age	− 30 years	17 (50.0)	60.7 (53.6–67.9)	68.8 (63.6–72.7)	57.1 (51.4–65.7)	78.6 (75.0–85.7)	67.0 (60.7–69.6)
	+ 30 years	17 (50.0)	53.6 (40.5–66.7)	66.2 (55.8–71.4)	54.3 (40.0–68.6)	71.4 (57.1–78.6)	55.8 (52.7–68.8)
			*p* = 0.290	*p* = 0.218	*p* = 0.339	*p* = 0.079	*p* = 0.150
Parity	Primipara	9 (26.5)	67.9 (61.9–67.9)	68.8 (61.0–74.0)	60.0 (54.3–80.0)	82.1 (75.0–85.7)	68.8 (64.3–71.0)
	Multipara	25 (73.5)	53.6 (45.2–60.7)	67.5 (58.4–71.4)	54.3 (45.7–65.7)	71.4 (57.1–82.1)	60.7 (54.5–68.8)
			*p* = 0.188	*p* = 0.565	*p* = 0.246	*p* = 0.086	*p* = 0.140
Hospital admission (mother)	Yes	5 (14.7)	65.5 (56.0–67.9)	67.5 (64.9–72.7)	54.3 (51.4–80.0)	78.6 (75.0–82.1)	68.8 (64.3–69.2)
	No	29 (85.3)	54.8 (40.5–67.9)	68.8 (58.4–71.4)	57.1 (45.7–65.7)	75.0 (64.3–85.7)	62.1 (53.6–69.6)
			*p* = 0.241	*p* = 0.460	*p* = 0.377	*p* = 0.587	*p* = 0.244
Hospital admission (child)	Yes	4 (11.8)	67.9 (64.3–69.9)	73.4 (69.8–76.9)	80.0 (77.9–80.0)	80.4 (74.1–85.7)	72.1 (69.2–75.1)
	No	30 (88.2)	55.4 (41.7–66.4)	67.5 (58.8–71.4)	54.3 (47.1–62.9)	75.0 (58.9–84.8)	61.4 (53.8–68.8)
			*p* = 0.138	*p* = 0.096	*p* = 0.002	*p* = 0.272	*p* = 0.025
Days with asthma symptoms	< 1/week	25 (73.5)	60.7 (53.6–67.9)	68.8 (61.0–71.4)	60.0 (51.4–71.4)	75.0 (71.4–85.7)	67.0 (55.8–69.6)
	≥ 1/week	9 (26.5)	46.4 (38.1–58.3)	67.5 (51.9–72.7)	54.3 (45.7–57.1)	64.3 (57.1–85.7)	60.3 (49.1–64.7)
			*p* = 0.175	*p* = 0.908	*p* = 0.263	*p* = 0.419	*p* = 0.202
Visits to asthma doctor	None	14 (41.2)	53.6 (42.0–65.5)	66.2 (60.1–70.8)	54.3 (47.1–67.1)	75.0 (71.4–78.6)	60.5 (54.1–68.3)
	≥ 1/2 weeks	20 (58.8)	59.5 (51.5–68.2)	69.5 (59.7–71.8)	57.1 (51.4–67.1)	76.8 (57.1–85.7)	66.1 (55.1–70.6)
			*p* = 0.290	*p* = 0.500	*p* = 0.641	*p* = 0.743	*p* = 0.259

^1^Except for hospital admissions that were tested by Student’s t tests

### Quality of life and asthma control and severity

Asthma severity strongly affected QoL. Women with MSPA had significantly lower symptoms related, activity related, and emotional QoL as compared to IMPA women (Table 3). Only QoL related to environmental stimuli did not differ between severity groups.

**Table 3:** Median (and interquartile range) of asthma quality of life questionnaire (AQLQ) domain scores and total score in relation to asthma severity in pregnant women with asthma. *p* value from Mann–Whitney test

AQLQ domain	IMPA (n = 23)	MSPA (n = 11)	Total (n = 34)	*p* value
Symptoms	65.5 (53.6–69.0)	39.3 (33.3–51.2)	56.5 (45.5–67.9)	< 0.001
Activities	70.1 (63.6–73.4)	58.4 (51.9–67.5)	68.2 (60.1–71.4)	0.012
Emotional	62.9 (54.3–77.1)	45.7 (37.1–54.3)	57.1 (51.4–67.9)	0.001
Environmental stimuli	75.0 (69.6–83.9)	75.0 (60.7–85.7)	75.0 (65.2–85.7)	0.942
Total	68.8 (60.5–70.3)	52.2 (45.8–58.3)	63.6 (54.7–69.5)	< 0.001

IMPA, intermittent asthma/mild persistent asthma; MSPA, moderate and severe persistent asthma

Similar differences were found with respect to asthma control. Women that were well controlled had higher overall asthma related QoL (*p* = 0.001) that was particularly pronounced for activity related and emotional QoL (Table 4).

**Table 4:** Median (and interquartile range) of asthma quality of life questionnaire (AQLQ) domain scores and total score in relation to asthma control in pregnant women with asthma. *p* value from Mann–Whitney test

AQLQ domain	well controlled (n = 13)	partly/poorly controlled (n = 21)	Total (n = 34)	*p* value
Symptoms	66.7 (53.6–67.9)	53.6 (39.3–61.9)	56.5 (45.5–67.9)	0.076
Activities	71.4 (67.5–74.0)	63.6 (53.2–70.1)	68.2 (60.1–71.4)	0.020
Emotional	71.4 (62.9–80.0)	51.4 (40.0–57.1)	57.1 (51.4–67.9)	< 0.001
Environmental stimuli	75.0 (75.0–85.7)	71.4 (57.1–85.7)	75.0 (65.2–85.7)	0.232
Total	69.6 (67.9–71.0)	55.8 (52.2–64.7)	63.6 (54.7–69.5)	0.001

No statistically significant differences were found when we differentiated by the frequency of asthma medication or systemic corticosteroid use (Table 5), the number of days with asthma symptoms or doctors’ visits (Table 2).

**Table 5:** Median (and interquartile range) of asthma quality of life questionnaire (AQLQ) domain scores and total score in relation to regular asthma medication and systemic corticosteroid (CS) use in pregnant women with asthma. *p* value from Mann–Whitney test

Grouping by*	AQLQ domain				
	Symptoms	Activities	Emotional	Environmental stimuli	Total
*Regular medication*					
None (n = 13)	53.6 (46.4–71.4)	67.5 (61.0–71.4)	54.3 (51.4–68.6)	75.0 (67.9–78.6)	62.9 (55.8–69.2)
≥ 1/day (n = 20)	56.5 (44.0–63.4)	67.5 (57.8–71.4)	57.1 (50.0–67.9)	76.8 (57.1–85.7)	63.4 (54.0–69.0)
*p* value	0.785	0.842	0.730	0.785	0.870
*Systemic CS use*					
No (n = 26)	53.6 (42.9–67.3)	67.5 (60.4–70.8)	57.1 (51.4–62.9)	75.0 (66.1–82.1)	62.1 (54.0–69.0)
Yes (n = 7)	60.7 (59.5–68.5)	71.4 (59.7–74.7)	65.7 (45.7–77.1)	85.7 (60.7–85.7)	67.4 (60.3–72.5)
*p* value	0.241	0.277	0.549	0.410	0.307
*Total*					
n = 33	56.0 (45.2–67.9)	67.5 (59.7–71.4)	57.1 (51.4–68.6)	75.0 (64.3–85.7)	62.9 (54.5–69.2)

*For one patient medication data got lost

Multivariable regression analyses revealed that deterioration of symptoms related QoL is dominated by asthma severity, while for the emotional domain asthma control is the most important predictor. Both asthma severity and control play about equal roles for activity related QoL but they do not reach statistical significance. Increasing age was associated with reduced QoL from environmental stimuli. Total asthma related QoL was most strongly related to severity of asthma (Table 6).

**Table 6:** Standardized regression coefficients (ß) and *p* values from multivariable linear regression analysis of asthma related quality of live domains and total score on asthma severity and asthma control, with age, ethnicity, area of living and parity considered as confounders

Predictor	Domain				
	Symptoms	Activities	Emotional	Environmental stimuli	Total
	ß (*p* value)	ß (*p* value)	ß (*p* value)	ß (*p* value)	ß (*p* value)
Age	− 0.095 (0.592)	− 0.298 (0.138)	− 0.144 (0.407)	− 0.478 (0.031)	− 0.266 (0.112)
Ethnicity	0.132 (0.406)	0.213 (0.231)	− 0.191 (0.223)	− 0.067 (0.723)	0.097 (0.509)
Area of living	− 0.149 (0.382)	0.027 (0.885)	− 0.130 (0.435)	0.064 (0.751)	− 0.086 (0.583)
Parity	− 0.074 (0.676)	0.067 (0.734)	− 0.069 (0.689)	− 0.012 (0.953)	− 0.031 (0.851)
Asthma severity	− 0.582 (0.002)	− 0.317 (0.102)	− 0.307 (0.074)	0.132 (0.520)	− 0.468 (0.006)
Asthma control	− 0.001 (0.994)	− 0.244 (0.183)	− 0.446 (0.009)	− 0.339 (0.089)	− 0.241 (0.116)

## Discussion

Although very good asthma related QoL was rare, the majority of pregnant women had at least partly favorable QoL especially in symptoms, emotional and activities domains. Asthma control was significantly related to asthma related QoL. This is in line with a previous study in Australia. [18]. A reduced QoL in pregnant women with not well controlled asthma was also reported by Burgess et al. [19]. We found no relationship between asthma medication and asthma related QoL although asthma control was significantly associated with QoL especially with respect to emotional function. This could be due to undertreatment since about half of patients that took no asthma medication during the last couple of weeks were not well controlled.

There is strong evidence that adequate control of asthma during pregnancy can improve health outcomes for mothers and babies [18, 19]. Despite known risks of poorly controlled asthma, a large proportion (62%) of women had sub-optimal asthma control. This could be due to concerns about risks of pharmacological agents. Under these circumstanced it would be advantageous if additional strategies for asthma control were available. However, as a Cochrane review stressed [20], there is insufficient evidence to date for alternative pharmacological as well as non-pharmacological interventions for asthma in pregnancy. Another review of non-pharmacological interventions came to the same conclusion [21].

Systemic corticosteroids seem to significantly improve quality of life [22], however, only seven of our patients received systemic corticosteroids and although on average their QoL was higher, this difference was statistically not significant. Schatz et al. [7] reported asthma-specific QoL during pregnancy is related to asthma exacerbations and to perinatal outcomes. Asthma exacerbations are defined as asthma symptoms requiring a hospitalization, other unscheduled medical visits, or increased doses of medication. Health-related QoL may be useful to identify patients at increased risk for asthma exacerbation requiring emergency care. In another study, it has been shown that 63% of women whose asthma symptoms were poorly controlled did not use a controller medication during pregnancy, suggesting inadequate treatment of potentially severe asthma symptoms [23]. Results about the impact of asthma on QoL may be of value for the development of instruments for use in clinical practice [24]. Burgess et al. [19] reported that pregnant women who did not reach appropriate control of their asthma experience reduced QoL and are likely to have more negative perceptions about their condition. They believed that their illness had a greater effect on their emotions and their lives in general in accordance with our findings. Also Powell et al. [18] found higher overall asthma related QoL in pregnant women with well-controlled asthma.

The diminished QoL in women with not well controlled asthma is partly due to their impaired pulmonary function limiting their physical activity. Physical inactivity during pregnancy could undesirably affect maternal QoL and pregnancy outcome. Controlling asthma in gravidity is thus essential for the QoL of the mother and welfare of the fetus [25].

There is a lack of data on safety for most drugs taken in pregnancy, because pregnant women are often excluded from clinical trials. Thus, one of the most important issues for the future is to provide more evidence about safety and efficacy of asthma treatment in pregnancy in order to improve asthma control under these conditions [26].

We found no effect of mother’s age and parity on asthma related QoL, except for a reduced QoL associated with environmental stimuli with increasing age of mothers in the multivariate analysis (Table 6). This could be due to an increase in sensitivity towards environmental stimuli or a broadening of the spectrum of asthma triggers with increasing age. A survey (NHANES) showed that the rate of allergic sensitization did not vary significantly with age [27]. Hence it might be concluded that rather sensitivity to triggers increases while the spectrum of triggers remains constant.

A strong relationship between asthma severity and QoL was found that was more or less restricted to the symptoms domain and reflects the impact of impaired respiratory function on QoL. Our results confirmed the usefulness of QoL measurements focusing on the quantification of the individual’s functional status, in studying consequences of severity and control of asthma.

If patients are involved in self-management, including self-monitoring of symptoms and respiratory function, maintaining regular contact with medical practitioners and following asthma action plans it may be possible to improve asthma control. More work is needed to clarify the priorities of pregnant women themselves with regard to their asthma management in pregnancy. Further studies will be necessary to determine, if measuring asthma-specific QoL is also advantageous in guiding asthma therapy during pregnancy and if longitudinal monitoring of asthma-specific QoL during pregnancy is advantageous to prevent pregnancy and perinatal complications [28].

The study has some limitations. More than 1600 pregnant women were screened, but only 34 women were diagnosed with asthma. We had expected more than double this number from a pilot study, but the determined prevalence was at the lower range of reported prevalences. This was likely due to asthma having not been self-reported as compared to the pilot and some previous studies, but was physician diagnosed. Another limitation is the single time-point for measuring asthma related QoL in the 2^nd^ trimester.

## Conclusion

We found that QoL in pregnant women with asthma is related to asthma severity and asthma control, a result in line with earlier studies. However, still some additional significant relationships were detected that should be considered in establishing care programs for women with asthma. The relationship between mothers’ age and QoL revealed by multivariate analysis suggests an increased sensitivity to asthma triggers and counselling women in avoiding triggers should be implemented in patients’ care. As suggested by reduced symptoms related QoL in Iranian women living in villages, barriers for maintaining regular contact with practitioners should be addressed in pregnancy welfare programs in Iran.

## Data Availability

The data are available from the authors upon reasonable request.

## Abbreviations

ACQ: Asthma Control Questionnaire
AQLQ: Asthma Quality of Life Questionnaire
GINA: Global Initiative for Asthma
ACOG: American College of Obstetricians & Gynecologists
ACAAI: American College of Asthma, Allergy and Immunology
IMPA: Intermittent asthma and mild persistent asthma
MSPA: Moderate and severe persistent asthma
QoL: Quality of life
